# Intestinal Absorption and First-Pass Metabolism of Polyphenol Compounds in Rat and Their Transport Dynamics in Caco-2 Cells

**DOI:** 10.1371/journal.pone.0029647

**Published:** 2012-01-13

**Authors:** Zenghui Teng, Chengjun Yuan, Feng Zhang, Menglei Huan, Weidong Cao, Kangchu Li, Jingyue Yang, Dayong Cao, Siyuan Zhou, Qibing Mei

**Affiliations:** 1 Department of Pharmaceutics, School of Pharmacy, Fourth Military Medical University, Xi'an, People's Republic of China; 2 Cardiovascular Department, First Affiliated Hospital, Xi'an Jiaotong University, Xi'an, People's Republic of China; 3 Key Laboratory of Gastrointestinal Pharmacology of Chinese Materia Medica of the State Administration of Traditional Chinese Medicine, School of Pharmacy, Fourth Military Medical University, Xi'an, People's Republic of China; 4 Xijing Hospital, Fourth Military Medical University, Xi'an, People's Republic of China; 5 Department of Radiation Medicine, Fourth Military Medical University, Xi'an, People's Republic of China; University of Pecs Medical School, Hungary

## Abstract

**Background:**

Polyphenols, a group of complex naturally occurring compounds, are widely distributed throughout the plant kingdom and are therefore readily consumed by humans. The relationship between their chemical structure and intestinal absorption, transport, and first-pass metabolism remains unresolved, however.

**Methods:**

Here, we investigated the intestinal absorption and first-pass metabolism of four polyphenol compounds, apigenin, resveratrol, emodin and chrysophanol, using the *in vitro* Caco-2 cell monolayer model system and *in situ* intestinal perfusion and *in vivo* pharmacokinetic studies in rats, so as to better understand the relationship between the chemical structure and biological fate of the dietary polyphenols.

**Conclusion:**

After oral administration, emodin and chrysophanol exhibited different absorptive and metabolic behaviours compared to apigenin and resveratrol. The differences in their chemical structures presumably resulted in differing affinities for drug-metabolizing enzymes, such as glucuronidase and sulphatase, and transporters, such as MRP2, SGLT1, and P-glycoprotein, which are found in intestinal epithelial cells.

## Introduction

Plant polyphenols—a group of complex, naturally occurring compounds—are widely distributed throughout the plant kingdom and are therefore readily consumed by humans. Humans are exposed directly or indirectly to polyphenols in medicinal and industrial applications, and in recent years interest has increased in the potential cancer-preventative and therapeutic properties of plant polyphenols derived from diet [1]–[2]. Epidemiological evidence has long suggested that dietary intake of polyphenols, which are abundant in fruits and vegetables, can reduce the risk of cardiovascular disease and cancer, and even Alzheimer disease [3]–[5]. A large number of mechanisms of action have been investigated, including antioxidant properties and effects on enzymes and signal transduction pathways *in vitro* and *in vivo* systems [6]–[7].

One main problem with polyphenols is our limited understanding of the relationship between their chemical structure and their intestinal absorption characteristics and metabolic pathways, making it difficult to understand their observed poor bioavailability and also restricting their targeted structural modification. Research on intestinal absorption and first-pass metabolism are a prerequisite for understanding a possible causal relationship between intake of polyphenols and their proposed chemopreventive effect [8]–[9].

**Figure 1:**
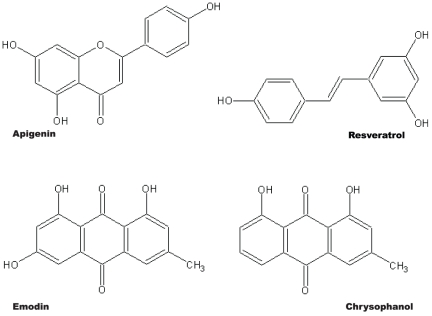
Chemical structures of the polyphenol compounds.

In the work presented here, we tested the hypothesis that both the hydroxyl and methyl groups of polyphenol compounds affect the metabolic stability and intestinal absorption characteristics in rat. To address this, we selected four polyphenol compounds that have different arrangements of hydroxyl and methyl groups: apigenin, resveratrol, emodin and chrysophanol (Figure 1). Emodin and chrysophanol each have one methyl group and differ only by one hydroxyl group—emodin has three and chrysophanol has two. Apigenin and resveratrol each have three hydroxyl groups but no methyl groups and have very different structures.

We analyzed the pharmacokinetics of these polyphenols in rat plasma and evaluated the contribution of the small intestine to the absorption and first-pass metabolism of polyphenols using an isolated small intestine perfusion system in rat, thereby allowing the direct estimation of intestinal absorption and transport, in addition to using Caco-2 cell line, a human intestinal epithelium model. We were thus able to investigate the relationship between the chemical structure and their intestinal absorption characteristics and metabolic pathways for these compounds.

## Results

### Pharmacokinetic analysis following oral administration of the polyphenols

**Figure 2:**
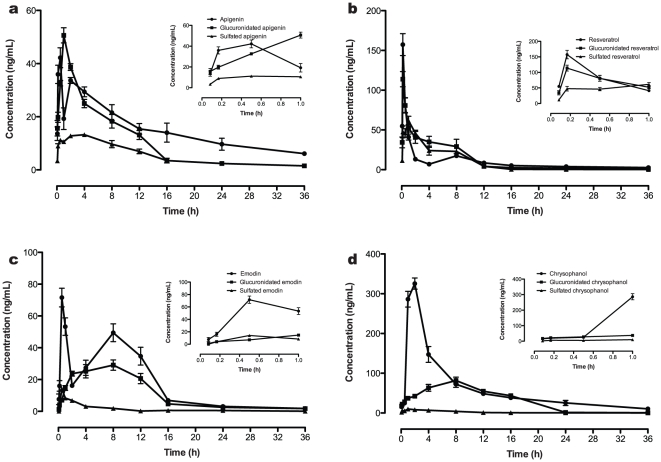
Time-dependent appearance of the polyphenol compounds and their metabolites in plasma after oral administration to rat. (A) apigenin, glucuronidated apigenin and sulphated apigenin, (B) resveratrol, glucuronidated resveratrol and sulphated resveratrol, (C) emodin, glucuronidated emodin and sulphated emodin, (D) chrysophanol, glucuronidated chrysophanol and sulphated chrysophanol, (mean ± SD, n = 6). (•) Parent polyphenols, (▪) glucuronidated polyphenols, (▴) sulphated polyphenols.

Following oral administration, the four polyphenols were rapidly absorbed into plasma. Figure 2 presents the plasma concentrations of each polyphenol as a function of time. Maximum concentrations (C_max_) for each polyphenol are observed in the following order: chrysophanol>resveratrol>emodin>apigenin. The double-peak phenomenon is observed in the concentration-time (CT) curve for apigenin, emodin and resveratrol, but not chrysophanol, which has greater much more higher absorption ratio, based on its area under the curve (AUC) value, and a longer elimination half-time (Figure 2). Of the polyphenols, the C_max_ of chrysophanol was the highest, and the AUC of resveratrol was the lowest. The intestinal absorption of intact emodin, apigenin and resveratrol was much faster than for chrysophanol, and the relevant pharmacokinetic parameters are listed in Table 1 which were determined by 3P97 software.

**Table 1:** Pharmacokinetic parameters of polyphenol compounds and their metabolites in rat plasma after oral administration.

Compounds	Pharmacokenitic parameters				
	*K* (h^−1^)	*T_1/2Ke_* (h)	*T_peak_* (h)	*C_max_* (ng/mL)	*AUC* (ng•h/mL)
Apigenin	0.16±0.02^1^	2.11±0.03	0.50±0.01	42±2	659±25
Glucuronidated apigenin	0.14±0.01	4.69±0.05	1.23±0.13	43±4	351±13
Sulfated apigenin	0.06±0.01	10.97±0.13	1.07±0.09	13±2	20±2
Resveratrol	0.38±0.03	1.82±0.05	0.17±0.02	157±13	98±7
Glucuronidated resveratrol	0.31±0.01	2.21±0.11	0.03±0.01	94±7	305±13
Sulfated resveratrol	0.16±0.02	4.09±0.23	0.22±0.03	56±3	350±24
Emodin	0.23±0.04	2.93±0.04	0.50±0.01	72±6	809±17
Glucuronidated emodin	0.14±0.03	4.83±0.17	4.48±0.14	28±2	387±3
Sulfated emodin	0.35±0.01	1.92±0.25	0.33±0.05	12±0.5	38±1
Chrysophanol	0.08±0.01	8.00±0.28	3.00±0.01	326±18	1721±57
Glucuronidated chrysophanol	0.13±0.02	5.23±0.47	6.45±0.24	67±1	1194±39
Sulfated chrysophanol	0.15±0.01	4.55±0.35	1.32±0.08	9±0.3	72±4

1 *mean* ± SD (all such values); *n* = 6.

LC/MS/MS and enzymatic hydrolysis with β-glucuronidase and sulphatase were used to identify and quantify the glucuronide and sulphate metabolites. After performing a full scan, individual ion peaks were selected and their fragmentation pattern was determined. Two of these were identified as glucuronidated (*m*/*z*+176) and sulphated (*m*/*z*+80) derivatives of the original compound (Figure S3). Quantitation was performed using multiple reaction monitoring of the deprotonated precursor ion and the related product ion after treatment with the β-glucuronidase and sulphatase, respectively. The resulting mass chromatographic peak areas of chrysophanol, resveratrol, emodin and apigenin increased significantly after treatment with β-glucuronidase and sulphatase (Figure S4) compared with the original compounds, indicating that glucuronidated and sulphated metabolites were indeed present in the plasma sample.

Within 1.2 h, the plasma level of apigenin glucuronides had a C_max_ value of 43±3 ng/mL (Figure 2), which is higher than that for apigenin sulphate. Resveratrol glucuronide rapidly reached its C_max_ value at 0.038 h. Compared to these compounds, the glucuronidated metabolites of emodin and chrysophanol did not reach their C_max_ values until 4.4 and 6.4 h later, respectively. The maximum concentration of resveratrol glucuronidates was reached much faster than that for resveratrol sulphates compared with other compounds whose peak level of sulphated metabolites appeared in the plasma first, ahead of the glucuronidated metabolites. Furthermore, only a small amount of the parent resveratrol was detected in the plasma, and most of it appeared to be present in the sulphated or glucuronidated metabolite form. The C_max_ and AUC of sulphated resveratrol were highest for the sulphated metabolites, and the AUC of chrysophanol glucuronides was higher than any other glucuronidated metabolite. The amount of derivatized metabolite detected, as a fraction of the original compound, was ranked as follows: resveratrol>apigenin>chrysophanol>emodin; thus the percentage of parent emodin in the plasma was much higher than that for the other polyphenols.

### Absorption and first-pass metabolism in the isolated intestine model

The rate of intestinal absorption and first-pass metabolism may also play an important role in determining the transport dynamics of the polyphenol compounds. To test the importance of intestinal metabolizing enzymes that are also involved in this metabolic process, we used an intestinal perfusion model in situ. Glucuronides and sulphates were identified and quantified as described in Methods. The amounts of derivatized compounds were determined indirectly by measuring the amounts of un-derivatized compounds following enzymatic cleavage of the derivatives by incubation of the luminal and vascular effluents (Table 2). Approximately 29% of the administered apigenin re-appeared at the vascular side—predominantly as free apigenin (16%)—but some apigenin glucuronides (9%) and sulphates (4%) were also detected. The main compound in the luminal effluent was the unmodified apigenin, with lesser amounts of apigenin glucuronides (13%) and sulphates (2%). For chrysophanol, 14% and 2% of glucuronides and sulphates, respectively, were detected at the vascular side; at the luminal side, 9% were chrysophanol glucuronides and 4% were sulphates. Glucuronidated and sulphated resveratrol was detected at the vascular side (16% and 24% respectively), and at the luminal side (5% and 7% respectively).

**Table 2:** Distribution of polyphenol compounds and their metabolites in the luminal and vascular compartments after perfusion experiments to rat small intestine.

Compounds	Luminal effluent		Vascular side	
	nmol	%	nmol	%
Apigenin^1^	813±34^3^	53.87^2^	247±19	16.39^2^
Glucuronidated apigenin	195±17	12.93^2^	131±14	8.70^2^
Sulfated apigenin	28±2	1.83^2^	57±8	3.79^2^
Resveratrol	536±27	35.37^2^	193±11	12.74^2^
Glucuronidated resveratrol	74±5	4.90^2^	243±10	16.04^2^
Sulfated resveratrol	105±7	6.90^2^	364±13	24.03^2^
Chrysophanol	559±57	36.82^2^	489±22	32.22^2^
Glucuronidated chrysophanol	131±12	8.63^2^	210±14	13.83^2^
Sulfated chrysophanol	66±8	4.34^2^	38±4	2.48^2^

1 polyphenol compounds (1500 nmol) were applied in perfusion experiments of 60 min, respectively.

2 refers to amount of delivered dose (%).

3 *mean* ± SD (all such values); *n* = 3.

**Figure 3:**
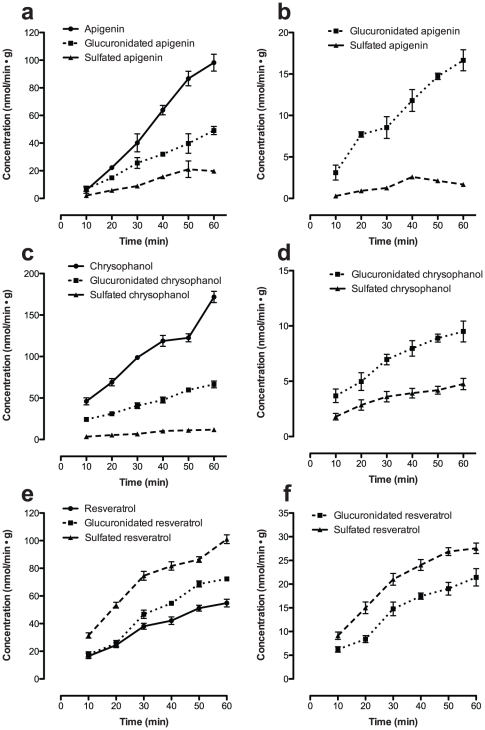
Time-dependent appearance of polyphenols and their metabolites in the luminal and vascular compartments after perfusion experiments with rat small intestine. (A) Apigenin, (C) chrysophanol, (E) resveratrol and their derivatives in the vascular compartments after perfusion experiments of chrysophanol with rat small intestine. (B) Apigenin, (D) chrysophanol, (F) resveratrol derivatives in the luminal compartments after perfusion experiments, (mean ± SD, n = 3). (•) Parent polyphenols, (▪) glucuronidated polyphenols, (▴) sulphated polyphenols.

Taken together with our earlier findings [10], following perfusion in the intestine, resveratrol was metabolized to the greatest extent, whereas only a small amount of emodin was metabolized. This is consistent with the AUC results of plasma concentrations following oral administration of the polyphenols: only a small amount of resveratrol was detected whereas emodin was detected in the greatest amounts. In addition, apigenin was metabolized to a similar extent as chrysophanol, although less parent compound was present on the vascular side. This is consistent with the AUC results of plasma concentrations following oral administration of apigenin and chrysophanol, with apigenin giving rise to a smaller AUC value. After first-pass metabolism, the percentage of resveratrol sulphates was higher than resveratrol glucuronides, and the majority of resveratrol metabolites were transferred to the vascular side. In contrast, the bulk of emodin, chrysophanol and apigenin metabolites were glucuronides that were detected in greater amounts at the luminal side compared to the vascular side (Figure 3). The control perfusion experiments, in which no polyphenols were added to the perfusion media, no polyphenols or their metabolites were detected.

### Transport of polyphenols through Caco-2 cells

**Figure 4:**
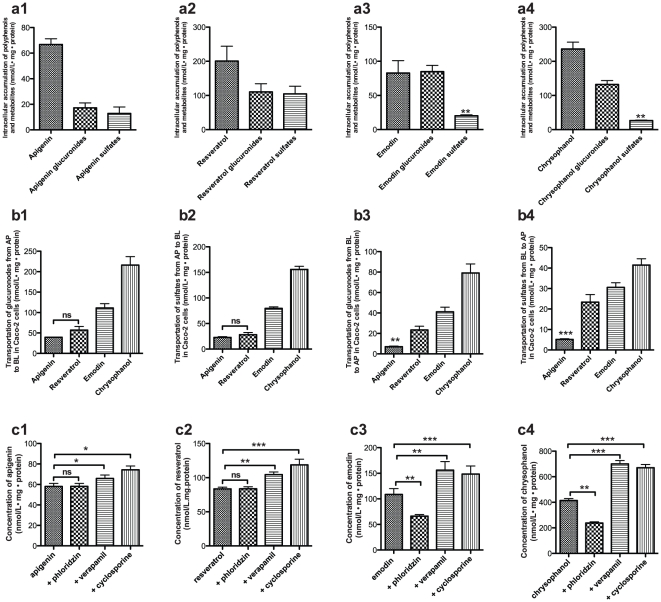
Transport and metabolism in Caco-2 cells. Intracellular accumulation of polyphenol compounds and their metabolites, (a_1_) apigenin and metabolites, (a_2_) resveratrol and metabolites, (a_3_) emodin and metabolites, (a_4_) chrysophanol and metabolites. Transport of glucuronides and sulphates from different directions in Caco-2 cells (nmol/L• mg • protein), (b_1_) glucuronides from AP to BL, (b_2_) sulphates from AP to BL, (b_3_) glucuronides from BL to AP, (b_4_) sulphates from BL to AP. The effect of different inhibitors (100 µM verapamil,100 µM cyclosporine A, 50 µM phloridzin) on the transport of polyphenol compounds in Caco-2 cells; (c_1_) apigenin, (c_2_) resveratrol, (c_3_) emodin, (c_4_) chrysophanol. (mean ± SD, n = 6).

We have previously shown that polyphenol compounds can be absorbed rapidly by Caco-2 cells [11]. After intracellular accumulation of these parent compounds, the C_max_ values were: chrysophanol>resveratrol>emodin>apigenin. The C_max_ of apigenin, chrysophanol and emodin was 10 min, but the intracellular accumulation of resveratrol increased in a time-dependent manner (Figure S5). After incubation with the Caco-2 cells for 1 h, the polyphenol compounds were metabolized by the phase II enzyme extensively. For emodin and chrysophanol, the predominant metabolites were still the glucuronides, but for resveratrol and apigenin there were no obvious differences between the amounts of glucuronides and sulphates (Figure 4a). This is in agreement with results from plasma and the isolated intestine samples.

Since Caco-2 is an in vitro model for the intestinal absorption, it is necessary to know how the parent compounds are transferred in Caco-2 cells in both directions firstly. So to observe the divergence in the transport processes of the polyphenol compounds and their metabolites, we monitored the directional movement of the intact parent compounds, glucuronide and sulfate derivatives in Caco-2 cells from apical to basolateral and from basolateral to apical directions, and the permeability values of the parent polyphenol and their metabolites were calculated from different direction. There were no changes in the permeability value for apigenin in either direction, but for emodin and chrysophanol the permeability value from BL to AP was much greater (by a factor of two) than that from AP to BL (*p*<0.01). The permeability value of resveratrol from BL to AP was lower than that from AP to BL (*p*<0.05) (Table 3). For all polyphenols, the metabolites had higher permeability values in the AP to BL direction than in the opposite direction, except for sulphated resveratrol, for which the permeability value was independent of the direction (Figure 4b).

**Table 3:** Permeability (Papp) of polyphenol compounds.

Polyphenols	P*_app_* (cm•s^−1^•10^−6^)	
	AP to BL	BL to AP
Apigenin	13.83±1.21^1^	12.91±2.54
Chrysophanol	10.81±2.34	27.38±4.19^2^
Emodin	9.52±1.45	24.45±3.22^2^
Resveratrol	11.29±3.45	7.86±2.67^3^

1 *mean* ± SD (all such values); *n* = 6.

2 Student's *t* test. Significantly different from the AP to BL direction, *p*<0.01.

3 Student's *t* test. Significantly different from the AP to BL direction, *p*<0.05.

To test the effects of the integral-membrane transporters on the absorption and transport of these polyphenol compounds, the intracellular accumulation of the polyphenol compounds was investigated in the absence or presence of verapamil, cyclosporine A, and phloridzin. The intracellular accumulation of apigenin and resveratrol clearly increased after pre-treatment with verapamil and cyclosporine A, but no changes were observed in the presence of phloridzin. We have previously shown that phloridzin, as a competitive inhibitor of SGLT1, can reduce the uptake of emodin and chrysophanol, It is interesting to note that phloridzin inhibits the uptake of emodin and chrysophanol, but not that of apigenin and resveratrol. Structurally the latter two compounds appear to bear some resemblance to phloridzin whereas the former two compounds do not. Here, however, verapamil and cyclosporine also increased the uptake of emodin and chrysophanol in a dose-dependent manner (Figure 4c). The inhibitors therefore appeared to dramatically affect the intracellular concentration of polyphenol compounds, indicating that the intracellular accumulation and transport processes of the polyphenol compounds in intestinal epithelial cells is regulated by these transporters.

## Discussion

Polyphenols are widely available as dietary supplements in pharmacies and health food stores [12]. Although the increasing evidence for the possible pharmacological effects of plant polyphenols on human health have been largely substantiated, concerns about their *in vivo* activity, especially regarding the relationship between their chemical structure and intestinal absorption, first-pass metabolism, or bioavailability remain unresolved. To address this issue, sensitive and reliable analytical methods capable of measuring polyphenols and their metabolism were developed and successfully used to determine oral bioavailability of polyphenols *in vivo*, pharmacokinetics studies using rats, absorption and first-pass metabolism mechanisms using *in situ* intestinal perfusion, and transport across Caco-2 cell monolayers.

All of the polyphenol compounds and their metabolites can be absorbed into the circulation rapidly after oral administration and exhibit different transport dynamics according to their chemical structures. The reason for high amounts of resveratrol absorption also originate from the fact, that this compound is a highly permeable substance. Furthermore, resveratrol absorption usually yields cis and trans isomers, and the amount of cis isomer formed also depends on the stability of the applied formulation. Plasma concentrations of glucuronidated metabolites were estimated to be 20-fold and 10-fold greater than that of the sulphated metabolites for chrysophanol and emodin, respectively. For apigenin and resveratrol, there are no clear differences between the fraction of glucuronidated and sulphated metabolites. It is therefore possible that the free hydroxyl groups of the latter polyphenols can give rise to very rapid derivatization by glucuronidation and sulphation, which arise from the hepatic or intestinal metabolic processes. In addition, the methyl groups of chrysophanol and emodin may hamper the production of the sulphated metabolite and increase the possibility of an interaction between the hydroxyl groups and glucuronide enzyme.

Systemic blood sampling cannot completely determine the contribution of the intestinal first-pass metabolism during the absorption of a drug or chemical, however. We therefore also used *in situ* intestinal perfusion in rat—a more reliable technique to investigate drug absorption potential, in combination with intestinal metabolism [13]. We used an isolated preparation of a vascularly and luminally perfused small intestine of a rat to assess the intestinal absorption and first-pass metabolism of these polyphenol compounds.

From these experiments, apigenin, resveratrol and emodin showed a higher percentage of parent drug in the luminal side and only a small amount was transferred into blood circulation after the first contact with enterocytes. There was no obvious difference for the concentration of parent chrysophanol between the luminal side and the vascular side, and the highest percentage of parent polyphenol was found in the vascular side. This is in agreement with that the observation that, compared to the other polyphenols, chrysophanol showed the highest AUC and C_max_ after oral administration.

In our current experiments with the isolated intestinal model, all of the parent compounds appeared to be metabolized extensively by the phase II enzymes in rat intestine. A fraction of the glucuronidated or sulphated polyphenols was absorbed into the blood, and the remainder was effluxed back to the luminal side. This experimental system allows the study of transepithelial transport and metabolism separately from processes taking place *in vivo* in the intestinal lumen, blood or liver. The presence of glucuronidated and sulphated metabolites in the vascular side of the small intestine of rats after perfusion of the polyphenols also shows that glucuronidation and sulphation of polyphenols occurs first in the enterocytes before further derivatization in the liver. These results strongly support previous evidence that most polyphenols are extensively metabolized and derivatized during transfer from the gut lumen to the serosal surface. It is noteworthy, however, that glucuronyl transferase or sulphate transferase may also play an important role in the first-pass metabolism of most polyphenols in the small intestine. This may ultimately reduce the bioavailability, and thus the efficacy, of orally administered polyphenols.

Furthermore, for apigenin and emodin, the total percentages of parent compounds and metabolites were estimated to be about two- to three-fold greater in the luminal side than in the vascular side. For chrysophanol and resveratrol, however, there were no clear differences in the concentration of parent compounds or metabolites between the luminal and vascular sides. We infer that the differences in chemical structure between these compounds and their metabolites is a factor that explains their different affinities for the transporters, including MRP2, SGLT1, and P-glycoprotein, all of which were found in the membranes of intestinal epithelial cells.

Because of its similarity to the human intestinal epithelium, the Caco-2 cell culture model has been widely used to determine intestinal transport and metabolism mechanisms for drug candidates and dietary chemicals [14]–[15]. Here, we used human Caco-2 cells to examine the relative transport of dietary polyphenols to gain insight into the mechanisms governing their absorption and metabolism processes in intestinal epithelial cells. We found that a specific transport system mediates the translocation of these polyphenol compounds across the apical membrane in Caco-2 cells. Phloridzin reduced the cellular uptake of emodin and chrysophanol in Caco-2 cells but had no effect on the intracellular absorption of apigenin and resveratrol. This suggests that emodin and chrysophanol can be absorbed and transferred via SGLT1. Both cyclosporine A and verapamil increased the uptake of these polyphenol compounds in Caco-2 cells, which means that MRP2 and P-glycoprotein were also the transporters for these compounds. In addition, this may partly be the reason for the double peak shown in the absorption of the polyphenol in plasma after oral administration. Usually, polyphenolic compounds metabolize in the enterocytes and are effluxed back into intestinal lumen by MRP-2/Pgp and in the lumen, these metabolites could be de-conjugated either by bacterial enzymes or enzymes from the desquamated cells, which could give rise for double peak. Also, this could occur in vivo in colon owing to metabolite excretion by bile and the re-absorption of parent compounds in the lower parts. Therefore, strategic application of intestinal P-glycoprotein, MRP2 and SGLT1 inhibitors may therefore modulate the oral therapeutic effect of these polyphenols.

The current study investigated the intestinal absorption and first-pass metabolism mechanism of four polyphenol compounds using the Caco-2 monolayer model, the rat *in situ* intestinal perfusion model, and *in vivo* pharmacokinetics in rat. Many studies have shown that the poor bioavailability of dietary polyphenols is dependent on the number of free hydroxyl groups, making them highly susceptible to glucuronidation and sulphation. This study also provides fundamental information on the relationship between the chemical structure of these polyphenols and their absorption characteristics and metabolic pathways in the intestine, and demonstrates that methyl groups of dietary polyphenols may result not only in a dramatic increase in their intestinal metabolic stability but also in a large improvement in their intestinal absorption—both of which should greatly increase their oral bioavailability. In addition, this study shows the usefulness of the intestinal perfusion model and intestinal Caco-2 cell monolayer, combined with *in vivo* pharmacokinetics studies, as effective methods to establish these properties. This investigation and with further structural modification, occurring in certain plants, provides insight into finding ways to improve the intestinal absorption of dietary polyphenols and the potential for the design of new therapeutic target drugs.

## Materials and Methods

### Ethics Statement

All procedures had the approval of the Animal Ethics Committee of the Fourth Military Medical University.

### Reagents and chemicals

Apigenin, chrysophanol, emodin, resveratrol and phloridzin were purchased from the National Institute for the Control of Pharmaceutical and Biological Products (Beijing, China) in the highest available purity (98%, as determined by high-performance liquid chromatography; HPLC). Due to their hydrophobicity and poor solubility in water, these polyphenol compounds were dissolved in dimethyl sulphoxide (DMSO) to give a solution of final concentration 0.1% v/v. Verapamil, cyclosporine A, β-glucuronidase and sulphatase were purchased from Sigma (St. Louis, MO, USA). All chemicals and reagents were of analytical grade or HPLC grade.

### Animals

Male Sprague-Dawley rats (70–110 days old) weighing between 270 g and 320 g were obtained from the experimental animal centre of the Fourth Military Medical University, Xi'an, China. The rats were subjected to fasting overnight, with free access to water before the day of experiment.

### Oral administration of polyphenols

The polyphenol compounds solution, prepared in 0.5% sodium carboxyl methyl cellulose (CMC-Na), were orally administrated to the rats using a dose of 50 µmol•kg^–1^. Blood samples were collected from the *vena abdominalis* under light ether anaesthesia at different times (0.08, 0.17, 0.5, 1, 2, 4, 8, 12, 16, 24 and 36 h). The samples were immediately centrifuged for 10 min at 7185 *g* to obtain plasma, which was then frozen at −70°C until required for analysis.

### In situ intestinal perfusion model

The rats were anaesthetized with an intra-abdominal injection of a mixture containing 40 mg•kg^–1^ sodium phenobarbital. The animals were then heparinized (90 U•kg^–1^) via the *vena caudalis*. The small intestine was prepared as described [16]–[17]. Briefly, a segment of intestine between 7 and 11 cm long was identified and separated and silicone tubing was placed inside both ends of the segment. The tube at the proximal side was connected to a peristaltic pump for luminal perfusion. A polyethylene cannula, connected to a peristaltic pump for vascular perfusion, was inserted into the superior mesenteric artery. The solutions on both sides were circulated with 95% O_2_ and 5% CO_2_ for the duration of the transport studies. Samples were obtained from the outlet of the mesenteric vein, and luminal aliquots were collected into preweighed microtubes every 10 min and stored at −70°C until needed for analysis.

### Cell experiments

Caco-2 cells, derived from human colorectal adenocarcinoma cells, were obtained from the American Type Culture Collection (Manassas, VA, USA) and maintained in plastic culture flasks (Corning Costar, Cambridge, MA, USA). The cells were cultured in Dulbecco's modified Eagle's minimal essential medium supplemented with 1% non-essential amino acids, 1% l-glutamine, 20% foetal bovine serum, 100 U•mL^–1^ penicillin, and 0.1 mg•mL^–1^ streptomycin, and were grown in a humidified atmosphere of 5% CO_2_ in air at 37°C (Figure S1). Cells were subcultured to 80% confluency [18].

For all cellular uptake studies of polyphenols, Caco-2 cells were seeded at a cell density of 6×10^5^ cells•cm^–2^ on six-well plastic plates. Cells were used 14 to 21 days after seeding. The culture medium was replaced with fresh medium 24 h before the uptake experiments. Polyphenol compounds (50 µM) were added to evaluate the uptake characteristics of Caco-2 cells. To determine the intracellular concentration of polyphenols, the cell lysate was obtained by subjecting the drug-containing cells to three freeze-thaw cycles in liquid nitrogen. Protein concentrations were measured using the Bradford method with bovine serum albumin as a standard.

For transport experiments, Caco-2 cells were seeded at a cell density of 1×10^5^ cells•cm^−2^ on Millicell Cell Culture Inserts (Millipore, Billerica, MA, USA). Cells were used 19 to 21 days after seeding, by which time the transepithelial electrical resistance value was >300 Ω•cm^–2^. The culture medium was replaced with fresh medium 24 h before the transport experiments. Polyphenol compounds (50 µM) were added from the apical or basolateral side to evaluate the transport characteristics of the cells. To investigate the role of different transporters, Caco-2 cells were preincubated with verapamil (an inhibitor of P-glycoprotein), cyclosporine A (a dual inhibitor of P-glycoprotein and multi-drug resistance protein 2, MRP2), or phloridzin, (a selective inhibitor of sodium/glucose cotransporter 1, SGLT1) to investigate the absorptive and transport changes of the polyphenol compounds [19]–[20]. The samples were collected and stored at −70°C until needed for analysis.

### Sample preparation and quantitative analysis

Metabolite samples were analyzed after treatment with β-glucuronidase or sulphatase [21]–[22]. The samples (0.2 mL) were extracted with 1 mL ethyl acetate by vigorous vortex mixing for 1 min, then centrifuged at 3000× *g* at room temperature for 10 min. The supernatants were evaporated to dryness under N_2_, reconstituted in 100 µL of methanol, and analyzed for the presence of polyphenols and their derivatives using liquid chromatography coupled with tandem mass spectrometry (LC/MS/MS) using a Quattro Premier system operating under MassLynx MS Software, v4.1 (Waters Corporation, Milford, MA, USA). We developed a high-throughput and sensitive bioanalytical method to estimate the concentration of polyphenol compounds and their metabolites using LC and electrospray ionization MS/MS to generate negative ions [M-H]^–^. Quantitation was performed by multiple reaction monitoring of the deprotonated precursor ion and the related product ion for these polyphenols using the internal standard method with peak area ratio [23]–[24]. Collision-induced dissociation was achieved using argon as the collision gas. Standard solutions of the polyphenol compounds (1 µg•mL^–1^) were applied to optimize the detection conditions in the presence of mobile phase. The mass transitions were *m*/*z* 269.4→225.4 for emodin, *m*/*z* 253.3→225.2 for chrysophanol, *m*/*z* 268.9→116.8 for apigenin, and *m*/*z* 226.9→142.8 for resveratrol (Table S2, Figure S2). The polyphenols were separated on a reverse phase C_18_ column (Symmetry C18 column, 5 µm, 2.1×50 mm, Waters Corporation, Milford, MA, USA) using an isocratic mobile phase consisting of 10% of 0.1% aqueous acetic acid/90% of 0.1% acetic acid in acetonitrile (v/v) for emodin and chrysophanol, and 40% of 0.1% aqueous acetic acid/60% of 0.1% acetic acid in acetonitrile (v/v) for resveratrol and apigenin (Table S1). The column temperature and flow rate were 25°C and 0.2 mL⋅min^–1^, respectively.

## Supporting Information

Figure S1 **Caco-2 cells for uptake and transport studies.**
(EPS)

Figure S2 **MassPrecursor/product ion mass spectra of polyphenols.** (a1) Apigenin, m/z 268.7; (a2) Daughter of apigenin, m/z 268.5→116.8; (b1) Resveratrol, m/z 226.9; (b2) Daughter of resveratrol, m/z 226.9→142.8; (c1) Emodin, m/z 269.4; (c2) Daughter of emodin, m/z 269.1→2 25.1; (d1) Chrysophanol, m/z 253.5; (d2) Daughter of chrysophanol, m/z 253.4→225.2.
(EPS)

Figure S3 **Precursor/product ion mass spectrum of the polyphenol metabolites.** (a_1_) Glucuronidated apigenin precursor ion mass spectrum, *m*/*z* 446. (a_2_) Daughter ion of glucuronidated apigenin, *m*/*z* 446→268.6. (a_3_) Sulphated apigenin precursor ion mass spectrum, *m*/*z* 349.7. (a_4_) Daughter ion of sulphated apigenin, *m*/*z* 349.7→268.5. (b_1_) Glucuronidated resveratrol precursor ion mass spectrum, *m*/*z* 402.7. (b_2_) Daughter ion of glucuronidated resveratrol, *m*/*z* 402.7→226.8. (b_3_) Sulphated resveratrol precursor ion mass spectrum, *m*/*z* 306.9. (b_4_) Daughter ion of sulphated resveratrol, *m*/*z* 306.9→226.7. (c_1_) Glucuronidated emodin precursor ion mass spectrum, *m*/*z* 446. (c_2_) Daughter ion of glucuronidated emodin, *m*/*z* 446→269.6. (c_3_) Sulphated emodin precursor ion mass spectrum, *m*/*z* 349.7, (c_4_) Daughter ion of sulphated emodin, *m*/*z* 349.7→269.3. (d_1_) Glucuronidated chrysophanol precursor ion mass spectrum, *m*/*z* 429.5. (d_2_) Daughter ion of glucuronidated chrysophanol, *m*/*z* 429.5→253.7. (d_3_) Sulphated chrysophanol precursor ion mass spectrum, *m*/*z* 333.2. (d_4_) Daughter ion of sulphated chrysophanol, *m*/*z* 333.7→253.6.
(EPS)

Figure S4 **Multiple reaction monitoring chromatograms of compounds and internal standard after treatment with β-glucuronidase or sulphatase.** The retention times were different for the polyphenols after separation on a reversed-phase column with an isocratic mobile phase, apigenin (1.82 min), resveratrol (1.30 min), emodin (1.17 min) and chrysophanol (1.57 min), respectively. (a_1_) Apigenin, (a_2_) glucuronidated apigenin, (a_3_) sulphated apigenin, (b_1_) resveratrol, (b_2_) glucuronidated resveratrol, (b_3_) sulphated resveratrol, (c_1_) emodin, (c_2_) glucuronidated emodin, (c_3_) sulphated emodin, (d_1_) chrysophanol, (d_2_) glucuronidated chrysophanol, (d_3_) sulphated chrysophanol.
(EPS)

Figure S5 **Time course for the intracellular accumulation of apigenin and resveratrol in caco-2 cells.**
(EPS)

Table S1 **HPLC condition.**
(DOC)

Table S2 **Mass spectrum condition.**
(DOC)
