# Supplementary material for: Intestinal Absorption and First-Pass Metabolism of Polyphenol Compounds in Rat and Their Transport Dynamics in Caco-2 Cells
Source: PLoS One. 2012 Jan 13;7(1):e29647. doi: 10.1371/journal.pone.0029647 (PMC3258254; doi:10.1371/journal.pone.0029647)
Supplement: Table S1 — HPLC condition. (DOC) [file pone.0029647.s006.doc]

**Table S1** HPLC condition

| Compounds | A (acetonitrile) | B ( water ,  0.1% acetic acid) | Internal Standard |
| --- | --- | --- | --- |
| Apigenin | 40 % | 60 % | Resveratrol |
| Resveratrol | 40 % | 60 % | Apigenin |
| Emodin | 90 % | 10 % | Chrysophanol |
| Chrysophanol | 90 % | 10 % | Emodin |
