# Supplementary material for: Intestinal Absorption and First-Pass Metabolism of Polyphenol Compounds in Rat and Their Transport Dynamics in Caco-2 Cells
Source: PLoS One. 2012 Jan 13;7(1):e29647. doi: 10.1371/journal.pone.0029647 (PMC3258254; doi:10.1371/journal.pone.0029647)
Supplement: Table S2 — Mass spectrum condition. (DOC) [file pone.0029647.s007.doc]

**Table S2** Mass spectrum condition

| Compounds | Precursor | Daughter | Dwell  time(s) | Capillary voltage(kV) | Cone voltage(V) | Collision energy(eV) |
| --- | --- | --- | --- | --- | --- | --- |
| Apigenin | 268.9 | 116.8 | 0.4 | 3.5 | 65 | 30 |
| Resveratrol | 226.9 | 142.8 | 0.4 | 3.5 | 40 | 30 |
| Emodin | 269.4 | 225.4 | 0.4 | 3.5 | 55 | 25 |
| Chrysophanol | 253.3 | 225.2 | 0.4 | 3.5 | 50 | 28 |
